# Established and Emerging Biomarkers in Cutaneous Malignant Melanoma

**DOI:** 10.3390/healthcare2010060

**Published:** 2014-01-14

**Authors:** Stamatina Verykiou, Robert A Ellis, Penny E Lovat

**Affiliations:** 1 Dermatological Sciences, Institute of Cellular Medicine, Newcastle University, Newcastle upon Tyne, NE2 4HH, UK; E-Mails: s.verykiou@newcastle.ac.uk (S.V.); robert.ellis2@newcastle.ac.uk (R.A.E.); 2 The James Cook University Hospital, South Tees Hospitals NHS Foundation Trust, Middlesbrough, TS4 3BW, UK

**Keywords:** cutaneous melanoma, malignancy, biomarkers

## Abstract

In an era of personalized medicine, disease specific biomarkers play an increasing role in the stratification of high-risk patient groups. Cutaneous malignant melanoma is the most deadly form of skin cancer with an ever-increasing global incidence, especially in patients under 35-years of age. Despite the excellent prognosis for patients diagnosed with early stage disease, metastatic disease still carries significant overall mortality. Biomarkers aim not only to identify high-risk patients, but also to provide potential therapeutic targets for differing patient subgroups. Furthermore, accessibility to tissue samples from a range of disease stages in malignant melanoma, unlike most other solid tissue tumours, provides the unique opportunity to explore the biology of tumour progression that may be relevant in the biology of cancer as a whole. Over the past decade, there have been major advances in targeted therapies, providing new avenues and hope to patients with this devastating disease. This review will focus on most up to date histological, serological and molecular biomarkers in malignant melanoma.

## 1. Introduction

A biomarker describes “any measurable diagnostic indicator that is used to assess the risk or presence of disease” [1]. There are various sub-types of biomarkers, but current interest in melanoma is largely focused towards the discovery of urgently required prognostic and predictive markers. Current prognostic biomarkers in malignant melanoma are based on the American Joint Committee on cancer (AJCC) criteria and, although they constitute some of the well-established biomarkers in solid tumours, are still unable to predict the subgroup of patients who will eventually progress to metastatic disease. 

Prognostic markers are able to stratify patients according to eventual outcome, and are used clinically to determine whether, for instance, a patient should receive adjuvant therapy following a diagnosis of melanoma [2]. The addition of a prognostic biomarker to the clinician’s repertoire thus allows patients to be triaged at the time of diagnosis and initial surgery. Those patients deemed at higher risk of metastases could be started on adjuvant therapies at an earlier stage than is currently possible, thus potentially decreasing the number of patients with untreatable metastatic disease [3].

Predictive biomarkers are able to indicate which patients would be likely to benefit from which treatment [2]. The importance of predictive biomarkers continues to grow in line with a trend towards personalized targeted treatment—a current example of a predictive biomarker is *BRAF* mutational status. Only patients with *BRAF*^V600E^ (and possibly *BRAF*^V600K^) show any benefit from the new class of BRAF inhibitors, namely Vemurafenib [4]. *BRAF* mutational status, however, cannot be used as a diagnostic or prognostic biomarker as mutations are also present in benign naevi, and although those melanomas with a *BRAF* mutation are more likely to develop regional metastases, there is no evidence of any effect on overall mortality [5].

In 2005 a commentary was released on behalf of the National Cancer Institute—European Organisation for Research and Treatment of Cancer (NCI-EORTC) outlining “Reporting Recommendations for Tumour Marker Prognostic Studies (REMARK)”. The overarching aim of these guidelines was to encourage transparent and complete reporting of biomarker studies so that appropriate conclusions can be drawn from their results. This document gives guidance on preferred methods for data analysis and presentation that allow its goals to be achieved when preparing work for publication, thus allowing a more robust comparison to be made between trial results [2]. 

The standard clinical method for melanoma diagnosis and stratification is based on immunohistochemistry (IHC). As such, a large number of potential biomarkers have been assessed using IHC as a readily available and clinically relevant methodology. An extremely comprehensive review that encompasses a wider range of IHC based protein biomarkers in melanoma that can be encompassed in this review, was undertaken by Gould Rothberg *et al.* in 2009 [6] and subsequently updated in 2010 [3]. These meta-analyses revealed 101 proteins that are good candidates for prognostic discrimination in melanoma. These proteins were involved in a range of tumour capabilities such as tissue invasion and metastasis, growth signalling and immunocompetence. 

Unfortunately, many tumour marker studies have not been reported in a rigorous fashion, and often lack sufficient information to allow adequate assessment of the quality of the study or applicability of results. Guidelines have been introduced to recommend elements and formats for presentation with the objectives of facilitating evaluation of the appropriateness and quality of study design, methods, analyses, and improving comparability of results across studies [2]. Five phases of biomarker development have been proposed. These include preclinical exploratory studies (Phase 1), clinical assay development for clinical disease (Phase 2), retrospective longitudinal repository studies (Phase 3), prospective screening studies (Phase 4), and cancer control studies (Phase 5) [7]. The REMARK guidelines introduced a more detailed algorithm in the design and reporting of biomarker development studies [2].

At present, no identified potential biomarker has undergone a large, rigorous, prospective trial with multivariate analysis that would allow it to be fully validated and developed for clinical practice. As such, there still remains an acute need for such markers in melanoma. This review aims to outline the current established biomarkers in melanoma, as well as reviewing the latest biomarkers of interest, and highlighted in the last few years.

## 2. Established Biomarkers in Melanoma

The current international standards for melanoma disease staging are based on the American Joint Committee on Cancer (AJCC) 2009 melanoma staging criteria. AJCC combines histological tissue variables, clinical characteristics as well as serological markers as prognostic biomarkers in order to stratify patients according to their prognosis. It must be noted that this system is still unable to identify those specific individuals that will develop metastases, and that the underlying biological relevance of these markers is still not fully elucidated [8].

### 2.1. Breslow Thickness

Alexander Breslow was the first person to report the role of tumour thickness as a biomarker predicting tumour progression [9]. In his initial study of 98 patients; tumour thickness, depth of invasion and cross sectional area was found to be associated with disease progression. Further studies have reinforced the clear prognostic importance of tumour thickness. The latest American Joint Committee on Cancer (AJCC) melanoma staging criteria confirmed the prognostic significance of Breslow thickness in a study cohort of 38,918 patients of varying melanoma disease stage. Breslow thickness was significantly associated with a decreased 10-year survival, from 92% in patients with T1 (≤1.00 mm thickness) melanomas to 50% in patients with T4 (>4.00 mm tick), reinforcing the use of Breslow depth as a principle stratifying tool of disease risk [10]. 

### 2.2. Tumour Ulceration

The enigmatic nature of prognostic biomarker biology in melanoma is most intriguing in the case of tumour ulceration. The presence of ulceration (the loss of epidermal integrity overlying the tumour) in a resected primary melanoma specimen has been shown to adversely affect outcome [10]. This, however, extends past outcomes based on the other physical characteristic of the primary resection alone. The 5-year survival of patients with N3 (melanoma deposits in >4 regional lymph nodes) disease with no ulceration in the primary tumour is 45.4%. This drops to only 29.2% if the primary melanoma was ulcerated [8], with no definitive explanation. It is not clear whether ulceration represents the effect of an intrinsically more aggressive tumour on the epidermis, or whether any tumour that becomes ulcerated results in an increased likelihood of spread.

The results of recent trials of adjuvant interferon seem to confirm this as yet undescribed aspect of ulceration in melanoma biology. Results of the EORTC 18952 and EORTC 18991 show that patients with nodal metastases after an ulcerated tumour have a more significant increase in progression free survival when treated with interferon-α when compared to matched controls with a non-ulcerated primary [11,12]. This suggests that the biological effects of ulceration are modified in some way by interferon-α. As a result of this prognostic biomarker, many clinical trials are now using ulceration of the primary tumour as a stratifying tool with adjuvant treatment being offered before evidence of any metastatic disease.

### 2.3. Mitotic Count

Primary melanoma proliferation as defined by the mitotic rate is another independent prognostic biomarker. Increased mitotic rate correlates with decreased survival and constitutes the second strongest predictor of survival following tumour thickness [10]. In melanomas with no mitoses present in the examined immunohistological specimen, the 10-year recurrence rate was as low as 1.8%, compared to lesions where ≥1 mitoses were present when the recurrence rate increased to 19.9% [13]. The presence of many mitotic figures indicates high metabolic cell activity and implies the ability of these tumours to enlarge and potentially metastasize [14]. 

## 3. Serological Biomarkers

Serological markers offer the advantage of easy, repeatable access to patient serum; in contrast to complicated surgical and radiological interventions so far required for the monitoring of patients with metastatic malignant melanoma. 

### LDH

Elevated levels of LDH accompany any disease characterized by cell destruction and may be used as a marker of tissue breakdown as a surrogate indicator of disease burden. Although LDH cannot be used as a diagnostic tool in patients with suspected metastatic melanoma due to low specificity, it may however be used as a monitoring tool for patients with advanced disease [15]. As such, elevated serum LDH has been included in the AJCC criteria as a prognostic biomarker in patients with stage IV disease. In patients with normal serum LDH levels, 1- and 2-year overall survival rates were 65% and 40% respectively, compared to only 32% and 18% when serum LDH levels were elevated [10]. In other studies, serum LDH >200 units was associated with poor prognosis in patients with malignant melanoma of stage III/IV [16]. According to the AJCC recommendations, serum LDH should be measured at diagnosis in patients with stage IV disease, and if found elevated patients are categorized as stage M1c (representing the least favourable prognosis) regardless of the site of distant metastases. Currently serum LDH is the only serological biomarker routinely used in clinical practice [17]. 

## 4. Emerging Biomarkers in Melanoma

### 4.1. Serological Biomarkers

S100 is expressed in cells of neural crest origin, as well as monocytes, macrophages and melanoma cells [18]. Currently, S100 is used extensively to differentiate melanocyte-derived lesions from other cutaneous tumours. S100B, a subunit of the S100 protein family, is detectable in the serum of melanoma patients and has been associated with tumour resistance and disease progression. In a study performed by Hauschild *et al.*, S100B was monitored in patients with metastatic melanoma receiving chemotherapy or immunotherapy. At 4 weeks, 78% of patients with tumour progression experienced an elevated S100B level, rising to 84% of patients by week 8. Patients who responded to systemic treatment showed stable or declining S100B levels compared to baseline, indicating the potential of S100B as a maker to monitor patients on treatment, indicating the need for re-staging and/or changes in therapeutic strategies in cases of persistently elevated levels during treatment [19].

Melanoma Inhibitory Activity (MIA) is a protein that interacts with extracellular matrix proteins. It is normally expressed in cartilage, but is also produced by malignant melanoma and to a lesser degree other tumours such as breast, colon cancer, and glioblastoma cells [20]. Studies have reported elevated serum MIA levels correlate with melanoma disease progression. Elevated MIA serum levels were found in 13 and 23% of patients with stage I and II disease respectively, compared to 100% of patients with stage III or IV disease [21]. Repeated follow-up measurements of MIA in 350 patients with a history of stage I or II melanoma revealed 32 patients who developed positive MIA values. 15 of these patients had developed metastases at time of the serological analysis, and one presented with metastatic disease six months later. In contrast, none of the patients with normal MIA serum levels developed metastases during the follow-up period, indicating a prognostic value of MIA detection in patients with melanoma [21].

Angiogenic factors and more specifically, vascular endothelial growth factor (VEGF) are important regulators of both normal and pathologic angiogenesis. In addition, VEGF excretion from tumour cells dramatically affects dendritic cell maturation from precursors; potential mechanism for the escape of tumours from the host immune system [22]. In a study performed by Ugurel *et al.*, serum levels of angiogenin, VEGF, bFGF, and IL-8 were significantly increased in patients with malignant melanoma compared to healthy controls. Elevated serum levels of VEGF, bFGF and IL-8 were strongly correlated with a poor overall and progression-free survival, suggesting that angiogenic serum factors VEGF, bFGF, and IL-8 are predictive markers for overall and progression-free survival in patients with malignant melanoma [23]. 

MicroRNAs (miRNAs) are small, non-coding RNAs regulating gene expression at the post-transcriptional level [24]. miRNAs have the advantage of direct analysis from patient blood as well as their stability against RNase digestion. So far, more than 2,000 miRNAs have been identified in the human genome and many have been associated with disease progression and risk of recurrence in patients with malignant melanoma. In a study performed by Friedman *et al.*, 355 miRNAs were screened in the serum of an initial cohort of 80 patients, later complemented with a “validation” cohort of another 50 patients. Five miRNAs (miR-150, -15b, -199a-5p, -33a, -424) successfully classified melanoma patients into high and low recurrence risk groups with significant separation of recurrence-free survival in both cohorts (*p* = 0.0036, *p* = 0.0093, respectively) [25]. Another study by Saldanha *et al.* analysed serum from 18 patients having diagnostic excisions, in whom the final histological diagnosis included benign nevus (*n* = 2), dysplastic nevus (*n* = 4), or stage 0–II primary melanoma (*n* = 12), stage III (*n* = 10) and stage IV (*n* = 4) melanoma patients, concentrating on the expression of miRNA-21. Analysis of all samples from controls to advanced melanoma showed a positive trend of plasma miRNA-21 (*p* < 0.0001). This trend was also seen with the disease-free and active melanoma groups (*p* < 0.0001), indicating that plasma miRNA-21 potentially reflects tumour burden and therefore might have a role monitoring disease activity and recurrence [26].

### 4.2. Circulating Tumour Cells

Detection of circulating cancer cells (CTC) has significant clinical potential as diagnostic markers of the presence of metastatic disease; as well as a role as a predictive marker for selecting appropriately targeted systemic therapy. Although malignant melanoma was the first solid tumour in which CTCs were detected [27], a lack of standardized methodology for isolation has reduced their use in clinical practice [28].

Recent studies have been undertaken isolating CTCs and correlating their expression with disease progression. A CellSearch platform (Veridex, Raritan, NJ, USA) has been used to isolate these subpopulations using antibodies against melanocyte surface markers (Melcam and high molecular weight melanoma-associated antibody (HMW-MAA)). In one clinical study, sera from 101 patients with metastatic cutaneous melanoma were analyzed prospectively. CTC number was determined using the CellSearch platform and melanoma kits in samples taken at baseline and serially during treatment. In 26% of patients with ≥2 CTCs at diagnosis the median overall survival was shorter when compared to those with ≤2 CTCs (2.6 *vs.* 7.2 months) [29]. Also, patients where CTC number remained at ≥2 CTCs during systemic treatment median overall survival was reduced compared to those patients in whom the CTC count remained ≤ 2; thus highlighting the potential of CTCs to act as both a prognostic biomarker and a marker of disease response [29,30]. 

Further, prospective studies measuring circulating melanoma cells in 230 patients with melanoma and 152 healthy controls identified two other potential prognostic serological CTC markers; MLANA, a melanocyte marker, and ABCB5, a stem-like cell marker [31]. Patients with melanoma were significantly more likely to express a melanoma cell marker in their blood (92%) compared to controls. Expression of two or more melanoma markers improved the positive predictive value to 86%, with a negative predictive value of 66%. These two markers, MLANA and ABCB5, had the greatest prognostic value, and were identified as statistically significant among patients who experienced disease recurrence during the study period, being expressed in 45% (MLANA) and 49% (ABCB5) of patients with recurrence [31].

### 4.3. Melanoma Initiating Cells

The identification of specific tumour sub-populations that have the ability to initiate and sustain tumour growth has led to the cancer stem cell (CSC) theory. The precise origin of melanoma however remains unknown, but recent studies suggest the accumulation of genetic and epigenetic mutations in melanocytes enables them to acquire stem cell-like capacities via somatic reprogramming [32]. Such stem-like properties allow these cells to self-renew, differentiate, and resist anoikis and apoptosis [33]. As such, the “cancer stem cell theory”, which has been linked to the development and progression of a number of malignancies, including hematopoietic and other solid tumour types [34], may also be associated with melanomagenesis. Different surface markers have been proposed to serve as putative CSC markers such as CD20, CD166, ABCG2, ABCB5 [34,35,36], with several studies focusing particularly on CD133 [35]. However CD133 is expressed by most of melanoma cell lines and it does not seem able to distinguish tumourigenic from non-tumourigenic cells [37]. A study by Klein *et al.* has correlated the immunohistochemical expression of different stem cell markers with disease progression. Tissue microarrays of normal tissue and 226 melanocytic lesions (71 banal nevi, 71 *in situ* and invasive melanomas and 84 metastatic melanomas) were investigated for immunohistochemical expression of CD166, CD133, and nestin. Primary and metastatic melanoma tissue expressed higher levels of CD166 (*p* = 0.005), CD133 (*p* = 0.003), and nestin (*p* = 0.03) than benign nevi. Nestin was the only marker demonstrating a statistical difference when comparing primary and metastatic melanoma (*p* = 0.05), with all cases of metastatic melanoma expressing at least one stem cell marker [38].

Markers focusing on functional properties of stem cells like CXCR6 are expressed by human melanoma cell lines and melanoma tissue. Studies comparing two parental human melanoma cell lines, one of primary tumour origin and one metastatic, demonstrated that the latter was more aggressive for tumour xenograft formation compared to primary melanoma cells. CXCR6+ cells resulted in larger tumour mass in a significantly shorter time period, as compared to unsorted cells. Furthermore, CXCR6− cells did not result in any significant, observable tumour mass, indicating that specific CXCR6+ subpopulation showed a strong self-renewal capability in a xenograft model [39].

Oct4 is an important transcription factor in stem cells that promotes de-differentiation of melanoma cells into CSC-like cells [40]. A study transiently expressing Oct4 in melanoma cell lines demonstrated that Oct4 expression can induce de-differentiation of melanoma cell into their CTC like progenitors and is associated with reactivation of other embryonic transcription factors. In addition these specific Oct4 positive subpopulations were more tumourigenic when injected into humanized mouse models, indicating that the presence of these subpopulations in tumours may signify a multi-drug resistant aggressive melanoma population [40]. Results to these studies are promising and could potentially give focus for novel therapeutic options, targeting these specific subpopulations. 

The CSC hypothesis provides an attractive cellular mechanism to account for the therapeutic resistance and aggressive behaviour exhibited by many tumours. CSC markers could potentially serve as biomarkers not only indicating a subset of patients likely to carry more aggressive tumours, but also indicating patients who are more likely to have a more drug resistant tumour subpopulation. 

## 5. Other Selected Biomarkers in Melanoma

Several marker molecules involved in genetic and molecular alterations have been identified, and their expression in primary melanoma has been correlated with prognosis. The identification of tissue specific markers that accurately identify patients with early disease who are at the highest risk of developing metastatic disease remains a high priority, and although no single marker is as yet able to consistently predict metastatic disease, several melanoma biomarkers have been associated with significant survival differences.

A number of studies have correlated gene expression with disease progression. A study by Bachmann *et al.* included 202 patients with malignant melanoma as part of a total of 700 patients which different types of cancer. EZH2, an important gene in cell cycle regulation, was associated with an aggressive tumour phenotype, with high levels of EZH2 correlating with reduced 5-year survival (EZH2-negative 71% patients disease free; EZH2-positive, only 48%) [41]. In a wide-ranging study performed by Winnepenninckx *et al.*, a total of 254 genes were associated with disease progression in 58 patients with metastatic melanoma. These initial results were validated using an immunohistochemical assay, with five genes MCM4, MCM3, MCM6, KPNA2 and geminin being associated with a significantly overall survival difference [42].

Examining samples at the protein level, a large prospective study by Weinlich *et al.* included 1,270 patients with malignant melanoma, analysed expression levels of metallothionein (MT) by IHC. Metallothioneins are intracellular proteins with high affinity for heavy metal ions. MT over expression in a variety of cancers is associated with resistance to anticancer drugs and overall poor prognosis. Results of this study associated over expression of MT in tumour cells of patients with primary melanoma with a higher risk for progression and reduced survival [43]. 

Tumour lymphagiomagenesis has been positively correlated with early lymph node metastases. Recent evidence highlights the tumour’s capability to induce lymphangiogenesis, mainly at the tumour-stroma interface, and correlates tumour lymphangiogenesis with increased incidence of sentinel lymph node metastases and disease-free survival. A study by Dadras *et al.* used immunohistochemistry of double immunostains for the lymphatic endothelial marker LYVE-1 and for the panvascular marker CD31 to stain samples from 18 primary melanomas with early lymph node metastasis and 19 from non-metastatic melanomas. The presence of intratumoural lymphatics was significantly higher in metastatic melanomas and correlated with poor disease-free survival compared to the non-metastatic melanoma cohort [44]. In addition, the presence of lymphatic tumour invasion was more strongly associated with lymph node metastases compared to presence of vascular invasion. A study performed by Doeden *et al.*, compared the frequency of lymphatic invasion *vs.* vascular invasion in melanoma sections from 94 patients using immunostains for the lymphatic endothelial markers D2-40 and LYVE-1 and the panvascular marker CD31. In a univariate analysis, lymphatic invasion was strongly associated with lymph node metastasis (*p* = 0.008). The presence of intratumoural lymphatics was associated with distant metastasis, whereas vascular invasion did not correlate with either lymph node or distant metastasis [45]. These studies indicate that tumour lymphangiogenesis can serve as a prognostic biomarker for lymph node metastasis in melanoma.

MicroRNAs (miRNAs) are noncoding, regulatory RNAs, involved in fundamental cellular processes such as differentiation, proliferation, and apoptosis [46]. The applicability of miRNA use in formalin fixed and paraffin embedded (FFPE) tissue has been the recent focus of several studies. One study compared miRNA expression patterns in FFPE *vs.* fresh frozen samples, using tissue from 15 melanocytic naevi. This study identified 84 miRNAs that were expressed in both types of samples and represented the miRNA profile of melanocytic naevi. These results demonstrated a high correlation in miRNA expression between paired FFPE and fresh frozen material [47]. One study used quantitative *in situ* hybridization (qISH) to evaluate the tumour suppressing properties of miR-205 in a cohort of 105, extended to another 206 primary FFPE melanoma samples. In this study, miR-205 expression was decreased in metastatic and primary melanomas compared to naevi, supporting the theory that miR205 acts as a tumour suppressor miRNA [48]. A different group used next-generation sequencing to carry out an in-depth analysis of miRNA transcriptome in biopsies of nevi, primary (>4.0 mm) and metastatic melanomas. Although several already known and novel miRNA targets were identified, a number of novel targets (miR-203, miR-204-5p, miR-205- 5p, miR-211-5p, miR-23b-3p, miR-26a-5p and miR-26b-5p) were decreased in melanomas compared to naevi [49]. 

These results reinforce the findings from previous studies indicating that miRNA analysis is feasible in FFPE tissue and could be used to study tissue specific biomarkers and highlight novel therapeutic strategies. 

Table 1 further summarizes many of the recently established or emerging independent prognostic biomarkers of interest in melanoma. 

**Table 1:** Selected historical and novel prognostic biomarkers.

Biomarker	Methods	Outcome	Reference
MITF	63 FFPE intermediate thickness melanomas stained by ICH and correlated with 50 month follow up	MTIF expression associated with longer DFS compared to no expression (187.90 +/− 13.41 months *vs.* 80.89 +/− 17.98 months)	Salti *et al.* [50]
Hsp90	Tissue microarrays of 414 nevi, 198 primary and 270 metastatic melanomas. Used automated quantitative analysis (AQUA) method of *in situ* Hsp 90 protein measurement	Although higher expression in metastatic compared to primary lesions (*p* < 0.0001), no association between high HSP90 and survival in the primary specimen cohort (*p* = 0.39)	McCarthy *et al.* [51]
CXCR4	Immunohistochemical expression of CXCR4 in 71 specimens of primary cutaneous melanoma with Breslow thickness of >1 mm	CXCR4 expression was correlated with an unfavorable prognosis with a median disease-free and overall survival of 22 and 35 months, respectively	Scala *et al.* [52]
HIF2α	Immunohistochemical expression in 46 nodular malignant melanomas	~175-month disease-specific survival HIF2α low: 87%; HIF2α high: 30%	Giatromanolaki *et al.* [53]
Nestin	Immunohistochemical study for nestin in 130 primary tumours and 32 nodal metastasis biopsy specimens	Nestin expression was associated with poor survival (log-rank test, *p* = 0.037),	Piras *et al.* [54]
HMGA2	Transcriptome profiling of 46 primary melanomas, 12 melanoma metastases, and 16 normal skin	HMGA2 expression is associated with disease-free survival (*p* = 0.004), overall survival (*p* = 0.008), and distant metastases-free survival	Raskin *et al.* [55]
Neuropilin-2	Immunohistochemical analysis in tissue microarray and histologic sections from samples of 42 primary melanomas, 30 metastatic melanomas, and 30 naevi	Expresision correlated with metastatic and primary melanoma compared to naevi (*p* < 0.0001)	Rushing *et al.* [56]
Metallothioneins (MT)	Immunohistochemical analysis of 1,270 primary and metastatic melanoma tissue	Overexpression of MT associated with higher risk for progression (117 of 167; 70.1%) and reduced survival (80 of 110; 72.7%)	Weinlich *et al.* [43]
NCOA3	Immunohistochemical analysis of tissue microarray in 343 primary cutanoues melanomas	High NCOA3 expression was associated with increased risk of death due to melanoma (31.9% *vs.* 18.5%;) and reduced DSS by (*p* < 0.030, log-rank test	Rangel *et al.* [57]
bFGF	Immunohistochemical analysis of 202 vertical growth phase cutaneous melanomas	bFGF +ve had a 59% 10-year survival, compared with 35% for patients with bFGF –ve vascular phenotype	Straume *et al.* [58]

## 6. Discussion and Conclusions

Malignant melanoma is a very enigmatic and heterogeneous cancer. Deregulation in oncogenes and tumour suppressors, as well as multiple molecular signals, are required for melanoma initiation and progression, leading to a range of interacting pathways. Attempts are ongoing to unravel this complex network, thus allowing the identification of novel genetic and molecular biomarkers, as well as potential therapeutic targets. 

Currently, the overriding prognostic indicator for patients with malignant melanoma is the disease stage at diagnosis. Although the AJCC staging system is able to predict outcomes in the majority of patients, there is still no accurate indicator to predict those individual patients with early stage disease at diagnosis who will still progress to metastatic disease. Therefore there is urgent need for identification of credible biomarkers that could accurately identify patients with early-stage disease who are at higher risk of metastasis. Disease specific biomarkers can stratify patients based on their response to treatment and therefore currently available treatment regimens could be used alone or in combination depending on individual patient and tumour characteristics. 

This review highlights not only the importance of established biomarkers, but also a number of tissue specific markers that have prognostic value in patients with malignant melanoma. Although the studies described in this review are promising, larger-scale studies need to be performed to evaluate the validity and usefulness of these markers and their applicability to daily clinical practice. In conclusion, the identification of novel biomarkers, representing distinct melanoma subpopulations and molecular signalling pathways, provides the foundations for the continued development of personalized medical interventions, be they diagnostic, prognostic or targeted therapies, well into the next decade.

## References

[1] Gogas H., Eggermont A.M.M., Hauschild A., Hersey P., Mohr P., Schadendorf D., Spatz A., Dummer R. (2009). Biomarkers in melanoma. Ann. Oncol..

[2] McShane L.M., Altman D.G., Sauerbrei W., Taube S.E., Gion M., Clark G.M. (2005). Statistics subcommittee of the NCI-EORTC working group on cancer diagnostics. Reporting recommendations for tumor marker prognostic studies (REMARK). J. Natl. Cancer Inst..

[3] Gould Rothberg B.E., Rimm D.L. (2010). Biomarkers: The useful and not so useful—An assessment of molecular prognostic markers for cutaneous melanoma. J. Invest. Dermatol..

[4] Chapman P.B., Hauschild A., Robert C., Haanen J.B., Ascierto P., Larkin J., Dummer R., Garbe C., Testori A., Maio M. (2011). Improved survival with vemurafenib in melanoma with BRAF V600E mutation. N. Engl. J. Med..

[5] Ellerhorst J.A., Greene V.R., Ekmekcioglu S., Warneke C.L., Johnson M.M., Cooke C.P., Wang L.E., Prieto V.G., Gershenwald J.E., Wei Q., Grimm E.A. (2011). Clinical correlates of NRAS and BRAF mutations in primary human melanoma. Clin. Cancer Res..

[6] Gould Rothbeg B.E., Bracken M.B., Rimm D.L. (2009). Tissue biomarkers for prognosis in cutaneous melanoma: A systematic review and meta-analysis. J. Natl. Cancer Inst..

[7] Alonzo T.A. (2005). Standards for reporting prognostic tumor marker studies. J. Clin. Oncol..

[8] Spatz A., Stock N., Batist G., van Kempen L.C. (2010). The biology of melanoma prognostic factors. Discov. Med..

[9] Breslow A. (1970). Thickness, cross-sectional areas and depth of invasion in the prognosis of cutaneous melanoma. Ann. Surg..

[10] Balch C.M., Gershenwald J.E., Soong S.J., Thompson J.F., Atkins M.B., Byrd D.R., Buzaid A.C., Cochran A.J., Coit D.G., Ding S. (2009). Final version of 2009 AJCC melanoma staging and classification. J. Clin. Oncol..

[11] Eggermont A.M., Suciu S., Testori A., Santinami M., Kruit W.H., Marsden J., Punt C.J., Salès F., Dummer R., Robert C. (2012). Long-term results of the randomized phase III trial EORTC 18991 of adjuvant therapy with pegylated interferon alfa-2b *vs.* observation in resected stage III melanoma. J. Clin. Oncol..

[12] Eggermont A.M., Suciu S., Testori A., Patel P., Spatz A. (2009). Ulceration of primary melanoma and responsiveness to adjuvant interferon therapy: Analysis of the adjuvant trials EORTC18952 and EORTC18991 in 2,644 patients. J. Clin. Oncol..

[13] Kesmodel S.B., Karakousis G.C., Botbyl J.D., Canter R.J., Lewis R.T., Wahl P.M., Terhune K.P., Alavi A., Elder D.E., Ming M.E. (2005). Mitotic rate as a predictor of sentinel lymph node positivity in patients with thin melanomas. Ann. Surg. Oncol..

[14] Francken A.B., Shaw H.M., Thompson J.F. (2004). The prognostic importance of tumor mitotic rate confirmed in 1,317 patients with primary cutaneous melanoma and long follow-up. Ann. Surg. Oncol..

[15] Jennings L., Murphy G.M. (2009). Predicting outcome in melanoma: Where are we now?. Br. J. Dermatol..

[16] Sirott M., Bajorin D., Wong G., Tao Y., Chapman P., Templeton M.A., Houghton A. (1993). Prognostic factors in patients with metastatic malignant melanoma. A multivariate analysis. Cancer.

[17] Sullivan R. (2013). The challenge of developing useful blood-based biomarkers in melanoma. Br. J. Dermatol..

[18] Cho K.H., Hashimoto K., Taniguchi Y. (1990). Immunohistochemical study of melanocytic naevus and malignant melanoma with monoclonal antibodies against S-100 subunits. Cancer.

[19] Hauschild A., Engel G., Brennerm W., Glaeser R., Moenig H., Henze E., Christophers E. (1999). Predictive value of serum S100B for monitoring patients with metastatic melanoma during chemotherapy and/or immunotherapy. Br. J. Dermatol..

[20] Bosserhoff A.K., Moser M., Hein R., Landthaler M., Buettner R. (1999). *In situ* expression patterns of melanoma-inhibiting activity (MIA) in melanomas and breast cancers. J. Pathol..

[21] Bosserhoff A.K., Kaufmann M., Kaluza B. (1997). Melanoma-inhibitory activity, a novel serum marker for progression of malignant melanoma. Cancer Res..

[22] Gabrilovich D.I., Chen H.L., Girgis K.R. (1996). Production of vascular endothelial growth factor by human tumors inhibits the functional maturation of dendritic cells. Nat. Med..

[23] Ugurel S., Rappl G., Tilgen W., Reinhold U. (2001). Increased serum concentration of angiogenic factors in malignant melanoma patients correlates with tumor progression and survival. J. Clin. Oncol..

[24] Bartel D.P. (2004). MicroRNAs: Genomics, biogenesis, mechanism, and function. Cell.

[25] Friedman E.B., Shang S., de Miera E.V., Fog J.U., Teilum M.W., Ma M.W., Berman R.S., Shapiro R.L., Pavlick A.C., Hernando E. (2012). Serum microRNAs as biomarkers for recurrence in melanoma. J. Transl. Med..

[26] Saldanha G., Potter L., Shendge P., Osborne J., Nicholson S., Yii N.W., Varma S., Aslam M.I., Elshaw S., Papadogeorgakis E. (2013). Plasma MicroRNA-21 is associated with tumor burden in cutaneous melanoma. J. Invest. Dermatol..

[27] Smith B., Selby P., Southgate J. (1991). Detection of melanoma cells in peripheral blood by means of reverse transcriptase and polymerase chain reaction. Lancet.

[28] Nezos A., Lembessis P., Sourla A. (2009). Molecular markers detecting circulating melanoma cells by reverse transcription polymerase chain reaction: Methodological pitfalls and clinical relevance. Clin. Chem. Lab. Med..

[29] Khoja L., Lorigan P., Zhou C., Lancashire M., Booth J., Cummings J., Califano R., Clack G., Hughes A., Dive C. (2013). Biomarker utility of circulating tumor cells in metastatic cutaneous melanoma. J. Invest. Dermatol..

[30] Karakousis G., Yang R., Xu X. (2013). Circulating melanoma cells as a predictive biomarker. J. Invest. Dermatol..

[31] Reid A.L., Millward M., Pearce R., Lee M., Frank M.H., Ireland A., Monshizadeh L., Rai T., Heenan P., Medic S. (2013). Markers of circulating tumour cells in the peripheral blood of patients with melanoma correlate with disease recurrence and progression. Br. J. Dermatol..

[32] Shakhova O., Sommer L. (2013). Testing the cancer stem cell hypothesis in melanoma: The clinics will tell. Cancer Lett..

[33] Girouard S.D., Murphy G.F. (2011). Melanoma stem cells: Not rare, but well done. Lab. Invest..

[34] Fang D., Nguyen T.K., Leishear K., Finko R., Kulp A.N., Hotz S., van Belle P.A., Xu X., Elder D.E., Herlyn M. (2005). A tumorigenic subpopulation with stem cell properties in melanomas. Cancer Res..

[35] Monzani E., Facchetti F., Galmozzi E., Corsini E., Benetti A., Cavazzin C., Gritti A., Piccinini A., Porro D., Antinami M. (2007). Melanoma contains CD133 and ABCG2 positive cells with enhanced tumorigenic potential. Eur. J. Cancer.

[36] Schatton T., Murphy G.F., Frank N.Y., Yamaura K., Waaga-Gasser A.M., Gasser M., Zhan Q., Jordan S., Duncan L.M., Weishaupt C. (2008). Identification of cells initiating human melanomas. Nature.

[37] La Porta C. (2009). Cancer Stem cells: A lesson from melanoma. Stem Cell Rev..

[38] Klein W.M., Wu B.P., Zhao S., Wu H., Klein-Szanto A.J., Tahan S.R. (2007). Increased expression of stem cell markers in malignant melanoma. Modern Pathol..

[39] Taghizadeh R., Noh M., Huh Y.H., Ciusani E., Sigalotti L., Maio M., Arosio B., Nicotra M.R., Natali P.G., Sherley J.L. (2010). CXCR6, a newly defined biomarker of tissue-specific stem cell asymmetric self-renewal, identifies more aggressive human melanoma cancer stem cells. PLoS One.

[40] Kumar S.M., Liu S., Lu H., Zhang H., Zhang P.J., Gimotty P.A., Guerra M., Guo W., Xu X. (2012). Acquired cancer stem cell phenotypes through Oct4-mediated dedifferentiation. Oncogene.

[41] Bachmann I.M. (2006). EZH2 expression is associated with high proliferation rate and aggressive tumor subgroups in cutaneous melanoma and cancers of the endometrium, prostate, and breast. J. Clin. Oncol..

[42] Winnepenninckx V., Lazar V., Michiels S., Dessen P., Stas M., Alonso S.R., Avril M.F., Ortiz Romero P.L., Robert T., Balacescu O. (2006). Gene expression profiling of primary cutaneous melanoma and clinical outcome. J. Natl. Cancer Inst..

[43] Weinlich G., Eisendle K., Hassler E., Baltaci M., Fritsch P.O., Zelger B. (2006). Metallothionein—Overexpression as a highly significant prognostic factor in melanoma: A prospective study on 1,270 patients. Br. J. Cancer.

[44] Dadras S.S., Paul T., Bertoncini J., Brown L.F., Muzikansky A., Jackson D.G., Ellwanger U., Garbe C., Mihm M.C., Detmar M. (2003). Tumor lymphangiogenesis, a novel prognostic indicator for cutaneous melanoma metastasis and survival. Am. J. Pathol..

[45] Doeden K., Ma Z., Narasimhan B., Swetter S.M., Detmar M., Dadras S.S. (2009). Lymphatic invasion in cutaneous melanoma is associated with sentinel lymph node metastasis. J Cutan. Pathol..

[46] Ambros V. (2004). The functions of animal microRNAs. Nature.

[47] Glud M., Klausen M., Gniadecki R., Rossing M., Hastrup N., Nielsen F.C., Drzewiecki K.T. (2009). MicroRNA expression in melanocytic nevi: The usefulness of formalin-fixed, paraffin-embedded material for miRNA microarray profiling. J. Invest. Dermatol..

[48] Hanna J.A., Hahn L., Agarwal S., Rimm D.L. (2012). *In situ* measurement of miR-205 in malignant melanoma tissue supports its role as a tumor suppressor MicroRNA. Lab. Invest..

[49] Kozubek J., Ma Z., Fleming E., Duggan T., Wu R., Shin D., Dadras S.S. (2013). In-depth characterization of microRNA transcriptome in melanoma. PLoS One.

[50] Salti G.I., Manougian T., Farolan M., Shilkaitis A., Majumdar D., Das Gupta T.K. (2000). Micropthalmia transcription factor: A new prognostic marker in intermediate-thickness cutaneous malignant melanoma. Cancer Res..

[51] McCarthy M.M., Pick E., Kluger Y., Gould-Rothberg B., Lazova R., Camp R.L., Rimm D.L., Kluger H.M. (2008). HSP90 as a marker of progression in melanoma. Ann. Oncol..

[52] Scala S., Ottaiano A., Ascierto P.A., Cavalli M., Simeone E., Giuliano P., Napolitano M., Franco R., Botti G., Castello G. (2005). Castello expression of CXCR4 predicts poor prognosis in patients with malignant melanoma. Clin. Cancer Res..

[53] Giatromanolaki A., Sivridis E., Kouskoukis C., Gatter K.C., Harris A.L., Koukourakis M.I. (2003). Hypoxia-inducible factors 1α and 2α are related to vascular endothelial growth factor expression and a poor prognosis in nodular malignant melanomas of the skin. Melanoma Res..

[54] Piras F., Perra M.T., Murtas D., Minerba L., Floris C., Maxia C., Demurtas P., Ugalde J., Ribatti D., Sirigu P. (2010). The stem cell marker nestin predicts poor prognosis in human melanoma. Oncol. Rep..

[55] Raskin L., Fullen D.R., Giordano T.J., Thomas D.G., Frohm M.L., Cha K.B., Ahn J., Mukherjee B., Johnson T.M., Gruber S.B. (2013). Transcriptome profiling identifies HMGA2 as a biomarker of melanoma progression and prognosis. J. Invest. Dermatol..

[56] Rushing E.C., Stine M.J., Hahn S.J., Shea S., Eller M.E., Naif A., Khanna S., Westra W.H., Jungbluth A.A., Busam K.J. (2012). Neuropilin-2: A novel biomarker for malignant melanoma?. Hum. Pathol..

[57] Rangel J., Torabian I., Shaikh L., Nosrati M., Baehner F.L., Haqq C., Leong S.P.L., Miller L.R., Sagebiel R.W., Kashani-Sabet M. (2006). Prognostic significance of nuclear receptor coactivator-3 overexpression in primary cutaneous melanoma. J. Clin. Oncol..

[58] Straume O., Akslen L.A. (2002). Importance of vascular phenotype by basic fibroblast growth factor, and influence of the angiogenic factors basic fibroblast growth factor/fibroblast growth factor receptor-1 and ephrin-A1/EphA2 on melanoma progression. Am. J. Pathol..

